# Supplementary material for: Tissue-specific and convergent metabolic transformation of cancer correlates with metastatic potential and patient survival
Source: Nat Commun. 2016 Oct 10;7:13041. doi: 10.1038/ncomms13041 (PMC5062467; doi:10.1038/ncomms13041)
Supplement: Supplementary Information — Supplementary Figures 1-9 [file ncomms13041-s1.pdf]

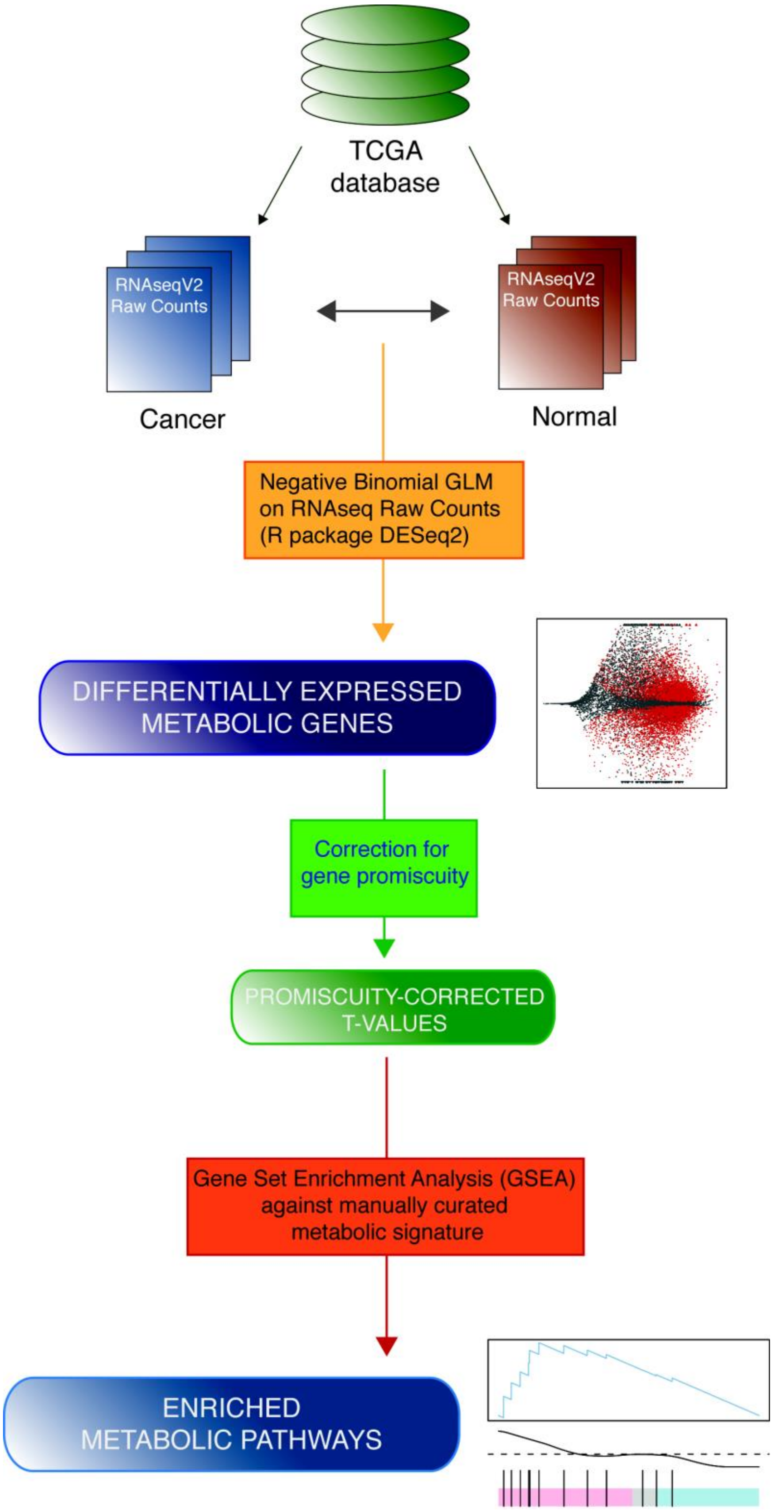

Supplementary Figure 1. Metabolic landscape of cancer discovery pipeline. RNAseq raw counts data of cancer and healthy tissue samples were downloaded from TCGA and differentially expressed metabolic genes were measured with the R package DESeq2. Genes associated with more than one pathway were subjected to correction for promiscuity and corrected t-values were used to perform gene set enrichment analysis (GSEA) against a manually curated metabolic gene signature.

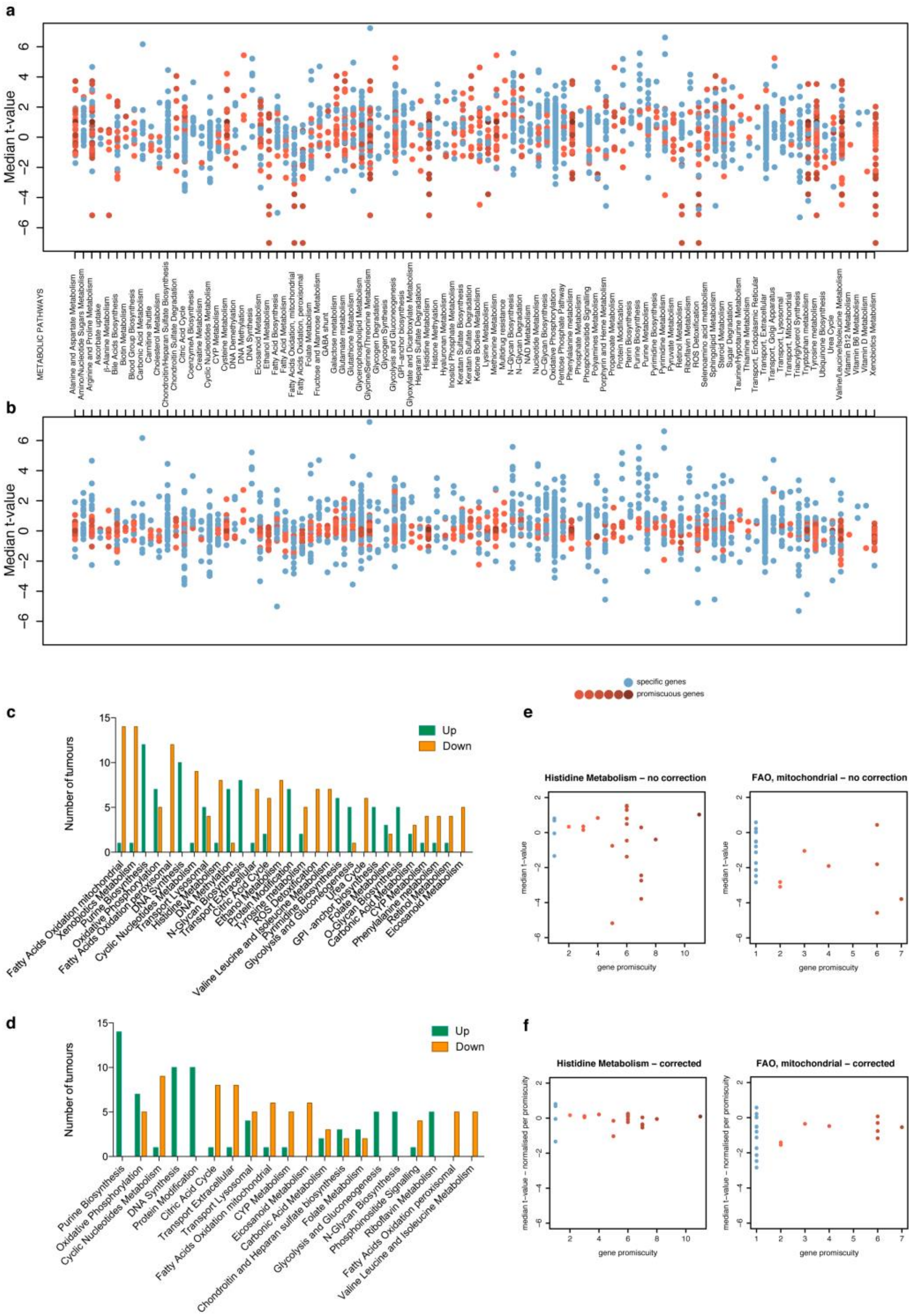

Supplementary Figure 2. Promiscuity of metabolic genes across the metabolic network. **a)** Non corrected and **b)** promiscuity-corrected median t-values of cancer vs normal for metabolic genes across 20 cancer types. Specific genes (blue dots) and promiscuous genes (red shading) are highlighted in order to compare the effect of promiscuity correction on differential gene expression. Frequency of enriched upregulated and downregulated metabolic pathways in cancers compared to normal before **(c)** or after **(d)** promiscuity correction. Metabolic pathways found enriched in at least 25% of cancers are shown. Median t-value of cancer vs normal for Histidine Metabolism and mitochondrial Fatty Acids Oxidation (FAO), before **(e)** and after **(f)** promiscuity correction. Promiscuous genes are highlighted in red shading to show the effect of correction for promiscuity on differential gene expression. FAO and Histidine Metabolism resulted as highly downregulated metabolic functions in several cancer samples compared to normal before promiscuity correction (**c**). Upon promiscuity correction, mitochondrial FAO remained among the top affected metabolic pathways in cancer samples, in line with a significant downregulation of specific, as well as promiscuous, genes associated with this pathway (**e**). Instead, Histidine Metabolism was not present in the top enriched metabolic functions after promiscuity correction, in accordance with a high proportion of significant and promiscuous, rather than specific, genes in this pathway (**e**).

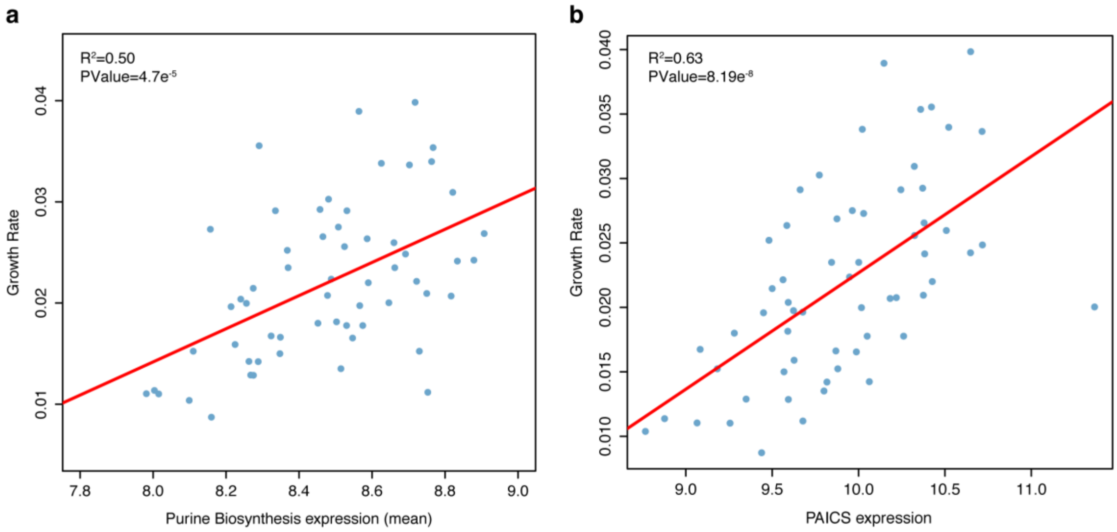

Supplementary Figure 3. Correlation of Purine Biosynthesis pathway with proliferation of cancer cell lines from the NCI-60 database. **a)** Scatter plot representation of Purine Biosynthesis genes (x axis) and growth rate (y axis). **b)** Scatter plot representation of expression of *PAICS* (x axis) and growth rate (y axis). Red lines indicate the linear trend between variables. Pearson correlation coefficient and p-values are indicated.

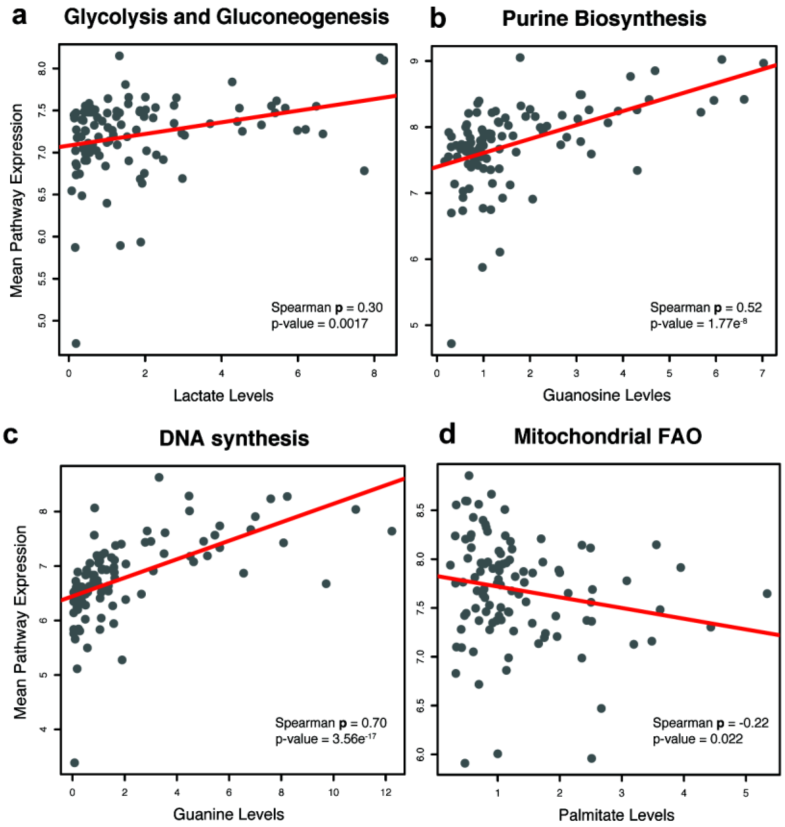

Supplementary Figure 4. Scatter plot indicating correlation between mean expression of indicated metabolic pathways and levels of (a) Lactate, (b) Guanosine, (c) Guanine, (d) Palmitate. Red lines indicate linear fit between mean pathway expression and metabolite levels.

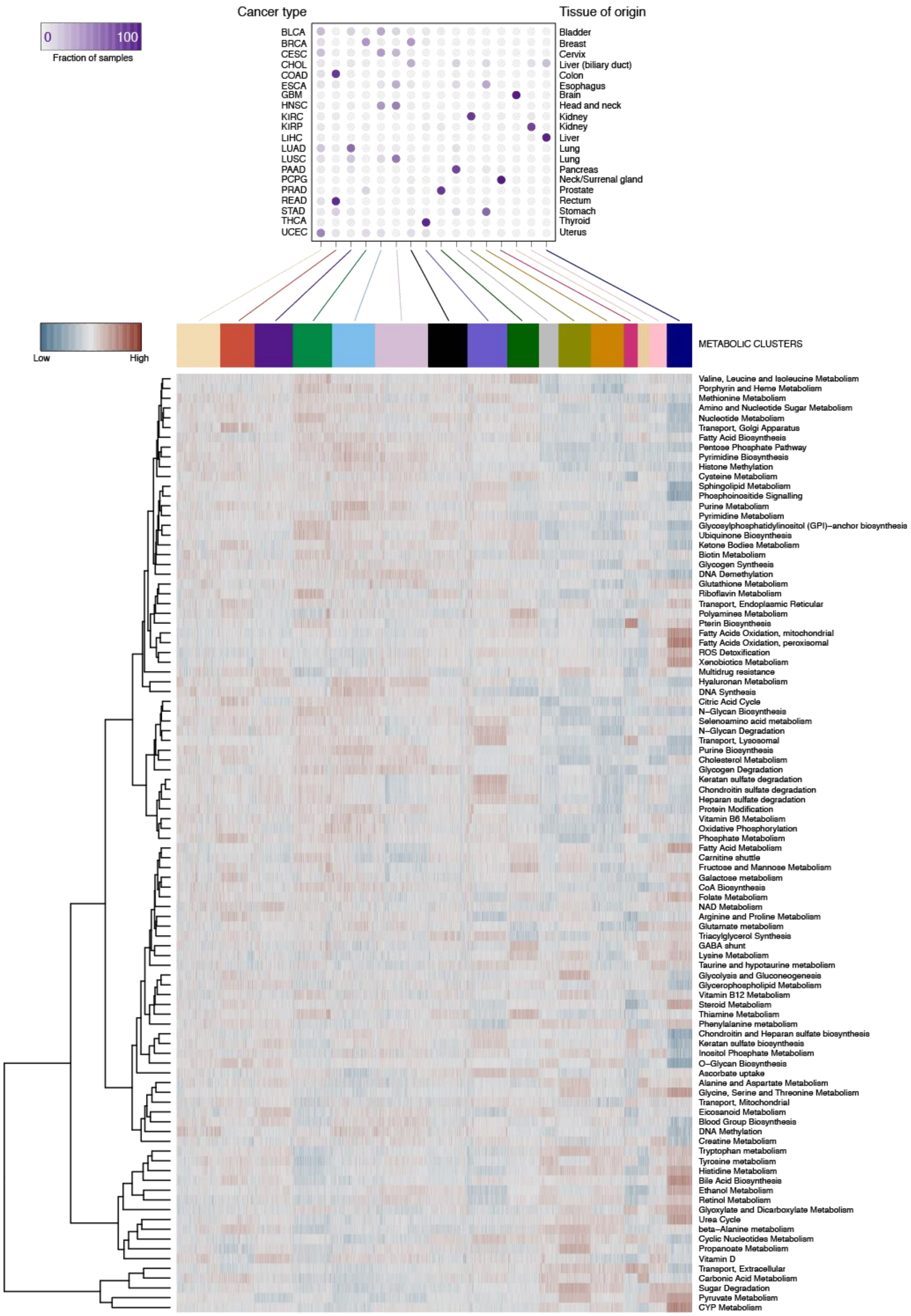

Supplementary Figure 5. Tissue-independent clustering of cancer samples based on expression of metabolic pathways. Heatmap representation of metabolic pathways in cancer samples only, divided into 16 clusters (top colored banner). Samples from each cancer type were overlapped on the 16 metabolic clusters and proportion of samples found in each cluster is reported in the top dot plot (purple shading indicates proportion of samples).

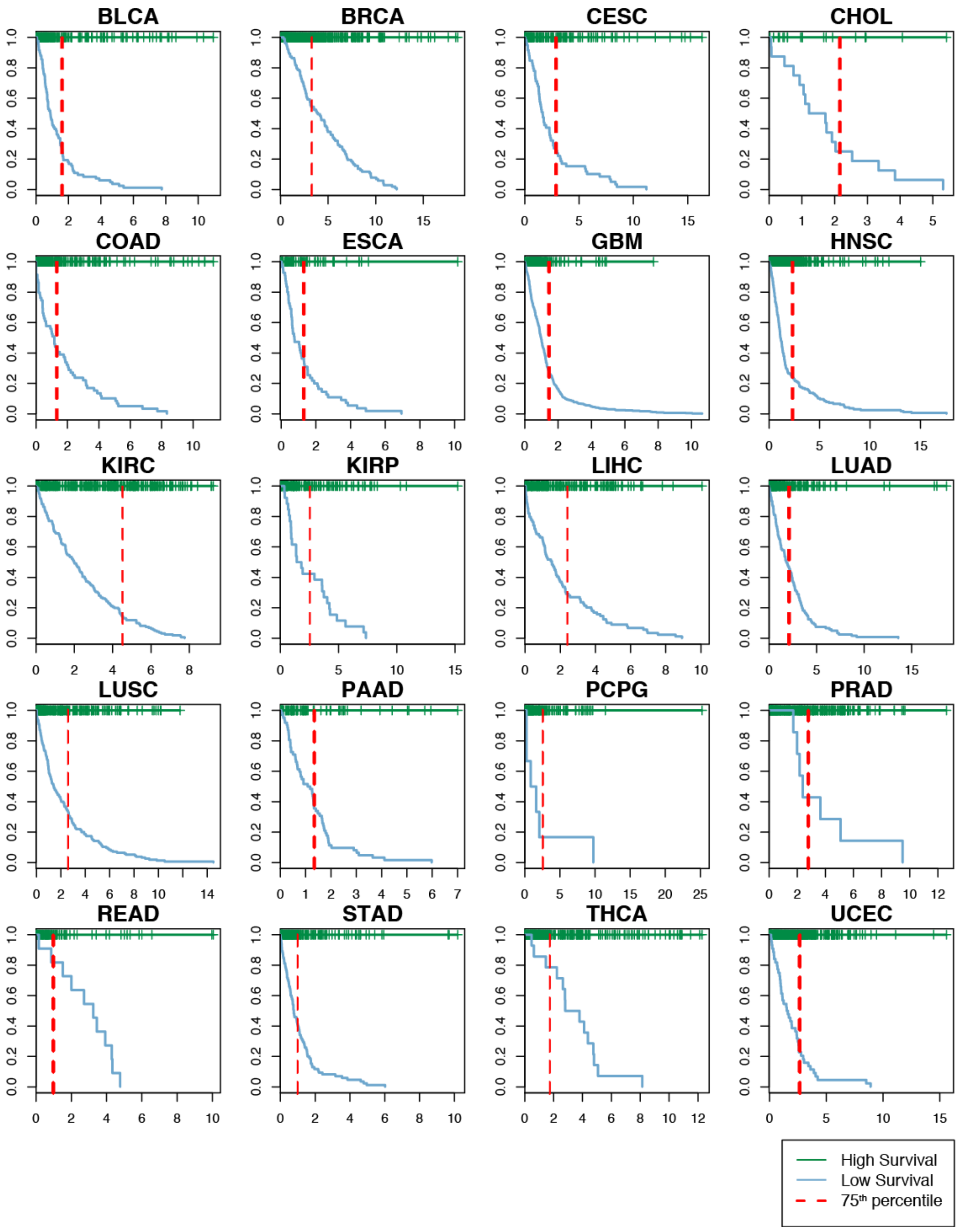

Supplementary Figure 6. Survival curves of High and Low survival patients. Red dashed line indicates 75th percentile of total study duration.

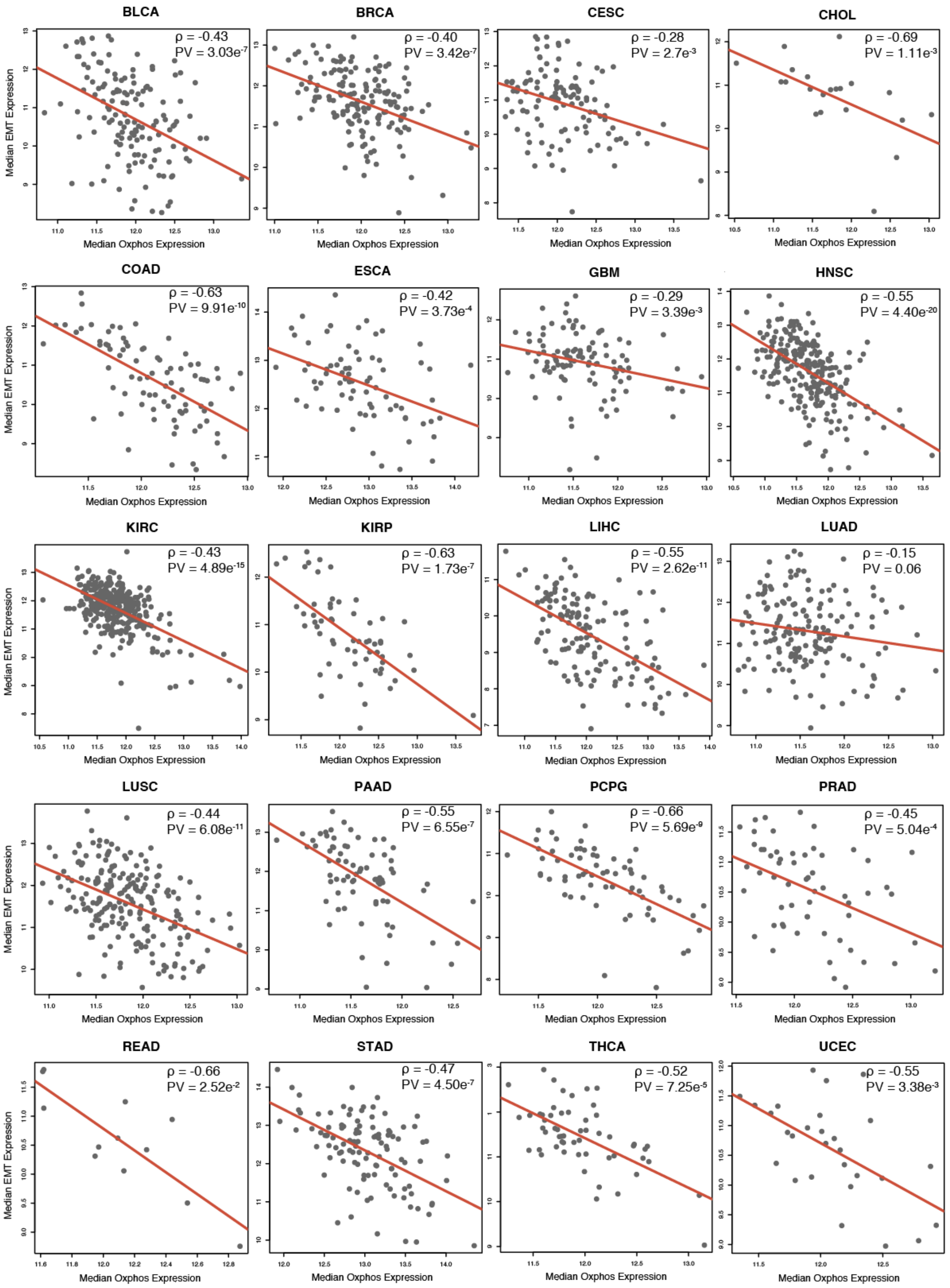

Supplementary Figure 7. Correlation between EMT induction and expression of OXPHOS across different cancers. Low and High survival patient cohorts of each cancer type were assessed for correlation between median EMT and median OXPHOS gene expression. Spearman correlation coefficient and p-values are indicated. Red lines indicate linear fit between EMT and OXPHOS.

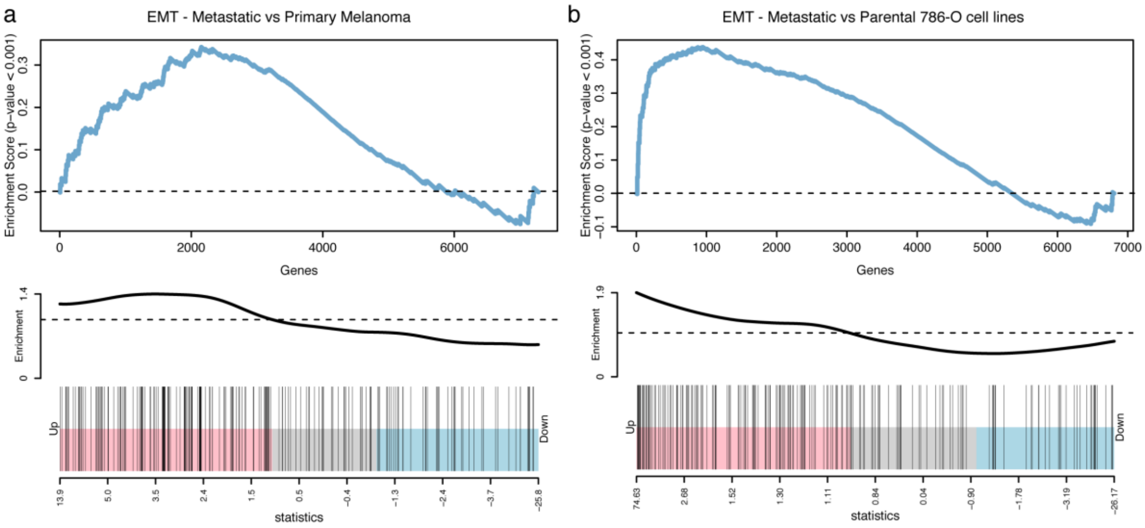

Supplementary Figure 8. Enrichment plot of Epithelial-to-Mesenchymal Transition (EMT) in metastatic vs primary melanoma cancer samples (a) and in metastatic vs parental 786-O cell lines (b).

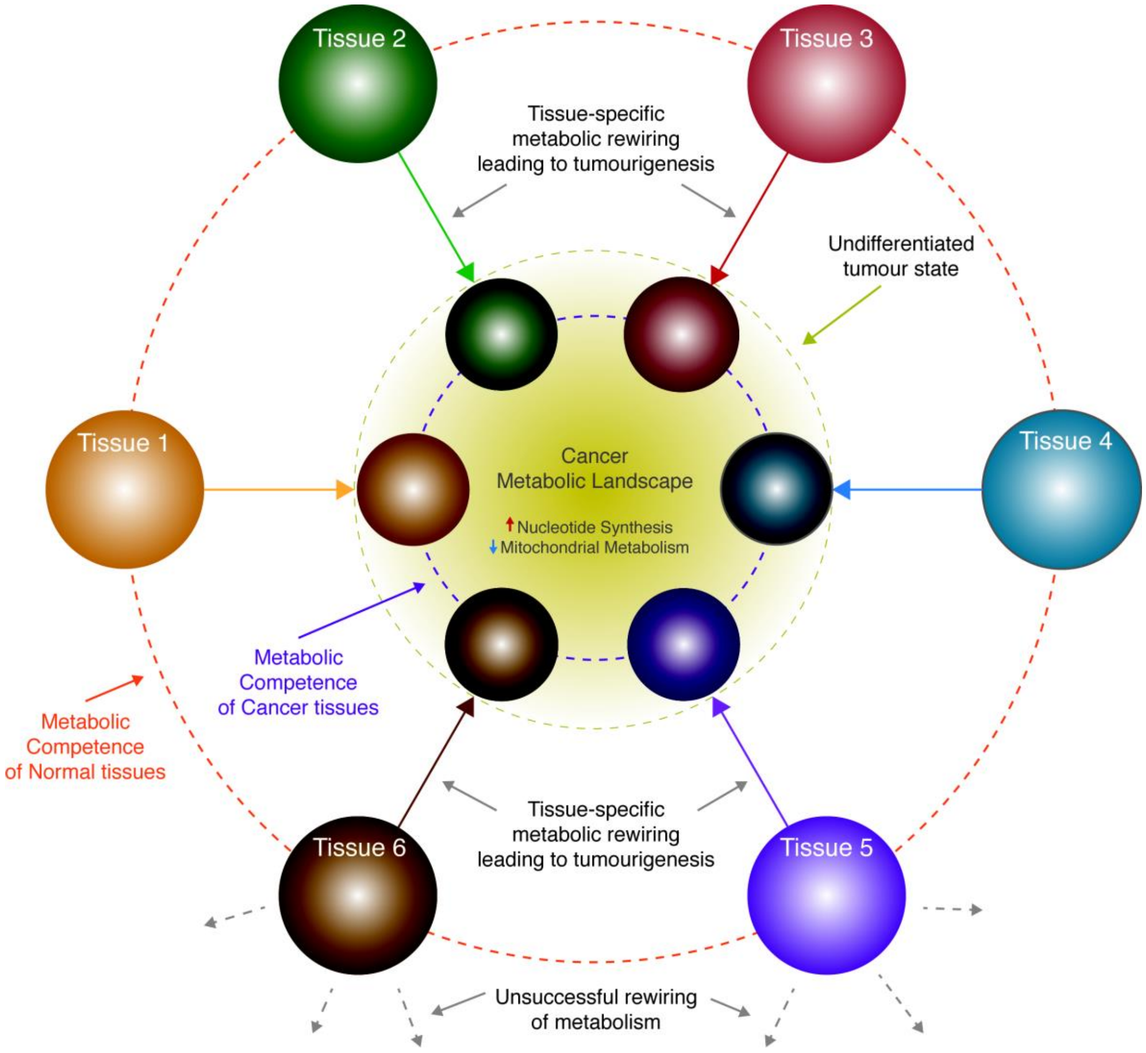

Supplementary Figure 9. The metabolic landscape of cancer. The metabolic rewiring (colored arrows) of individual tissue (external circles) undergoing transformation depends on the tissue of origin. Several attempts of transformation might take place from each normal tissue (grey dashed arrows), but only a metabolic rewiring that fulfils the requirements of the tissue of origin is selected and results into cancer formation. After transformation, even though cancer cells maintain tissue identity, they converge towards a common metabolic landscape characterized by expression of nucleotide synthesis genes and decreased expression of mitochondrial genes. Within this convergent landscape, cancers have lost part of the metabolic competence and cancers with decreased OXPHOS genes activity display poorer patient survival.
